# Mechanical and Thermoanalytical Study of Polypropylene Geomats Exposed in the Field and the Laboratory

**DOI:** 10.3390/ma16114148

**Published:** 2023-06-02

**Authors:** Jefferson Lins da Silva, Clever Aparecido Valentin, Marcelo Kobelnik, Gabriel Orquizas Mattielo Pedroso, Maria Alejandra Aparício-Ardila, Luiz Paulo Vieira de Araújo Júnior, Marta Pereira da Luz

**Affiliations:** 1 São Carlos School of Engineering (EESC), University of São Paulo, São Carlos 13566-590, Brazil; 2 Guaratinguetá Faculty of Engineering and Sciences (FEG), São Paulo State University (UNESP), Guaratinguetá 12516-410, Brazil; 3 Civil Engineering Department, Federal University of Sao Carlos, São Carlos 13565-905, Brazil; 4 Department of Dam Safety and Technology, Eletrobras Furnas, BR153, km 510, Zona Rural, Aparecida de Goiânia 74923-650, Brazil; 5 Industrial and Systems Engineering Postgraduate Program (MEPROS), Pontifical Catholic University of Goiás-PUC Goiás, Goiânia 74605-220, Brazil

**Keywords:** geomat, geosynthetics, erosion-control techniques, slope

## Abstract

A Hydroelectric Power Plant (HPP) presents erosion problems on reservoir slopes and margins. Geomats are a biotechnical composite technology increasingly used to protect soils against erosion. Survivability or durability of geomats is essential for successful application. This work analyses the degradation of geomats exposed in the field for more than six years. These geomats were used as erosion-control treatment in a slope at HPP Simplício in Brazil. The degradation of the geomats in the laboratory was also analysed through exposure in a UV ageing chamber for 500 h and 1000 h. Degradation was quantitatively evaluated by testing the tensile strength of the geomat wires and thermal tests such as thermogravimetry (TG) and differential scanning calorimetry (DSC). The results showed that the geomat wires exposed in the field had a greater decrease in resistance compared to the samples exposed in the laboratory. In the samples collected in the field, it could be observed that the degradation of the virgin sample occurred earlier than in the exposed samples, contrary to what was observed in the TG tests carried out with the samples exposed in the laboratory. The DSC analysis showed that the samples had similar behaviours for the melting peaks. This evaluation of the wires of the geomats was presented as an alternative to analysing the tensile strengths of discontinuous geosynthetic materials such as the geomats.

## 1. Introduction

Soil erosion consists of a process of separation, transport and deposition of sediments [1]. This process occurs through erosive agents such as rainfall and wind. Erosion caused by precipitation is the most critical detaching agent and is the effect of raindrops striking the soil surface, resulting in disaggregation due to the impact (splashing). Then, running water and wind are responsible for superficial flow (runoff) [1]. Problems caused by soil erosion and subsequent sedimentation are responsible for environmental impacts on slopes of reservoirs and silted-up rivers, especially in regions with intense rainfall and fine, cohesionless soils [2]. Therefore, it is crucial to prevent and control erosion to minimise its consequences.

Controlling erosion aims to curb or restrain soil’s gradual and sudden wear and tear [3]. A standard method is development of natural vegetation roots in combination with coverage materials, and the success of these techniques depends on growth of vegetation and the stability of the slope [4]. In such systems, rolled erosion-control materials hold the soil until permanent vegetation cover is established [5]. For effective erosion control, temporary and/or permanent erosion-control methods with different types of materials can be used, and the appropriate material depends on the site condition and the mechanical properties of the composite [6]. Permanent materials are subdivided into biotechnical composite technology when vegetation is reinforced [7] and armour systems when nonvegetated inert materials are installed.

In this context, geosynthetics with the functions of erosion-control products have become increasingly popular. The efficiency of different geosynthetic materials to restrain erosion has been carried out with laboratory and field evaluation [5,8,9,10,11,12,13,14]. A laboratory performance evaluation was conducted using mechanical properties tests [7,15] and rainfall erosion tests [10,11].

Geomats are a biotechnical composite technology and have been increasingly used to protect soils against erosion. Palmeira et al. [10] and Chen et al. [11] have shown that once vegetation is established, roots enter shallow soil and provide a structure, and that, over time, as vegetation increases, geomats decrease in importance. Therefore, survivability of geomats is essential for successful application, and it is expected that long-term, non degradable geomats have a minimum lifespan of 3 years and a maximum of 50 years. Li and Khanna [16] showed that the tensile strengths of geomats exposed for 3 years are the most important physical property for evaluating ageing effects, and their results showed that both natural and synthetic composites had strength reductions after exposure.

According to Li and Khanna [16], geomats can be subjected to different degradation mechanisms, such as oxidation, hydrolysis, physical degradation, chemical degradation, radioactivity and biological degradation. However, among these factors, ultraviolet degradation (UV) and temperature are considered the most critical. UV degradation can be evaluated in field studies and laboratory accelerated tests. Suits and Hsuan [17] reported that laboratory accelerated tests provide more consistent results, as the environment can be controlled. For these tests, UV test chambers are equipped with lamps that irradiate UV light, and samples are exposed to a controlled moisture and temperature ambient. Geosynthetic degradation evaluation is verified with a material’s change in physical and mechanical properties at different exposure times [18,19].

Recently, other fundamental analyses of macrostructural and microstructural degradation of geosynthetics have been evaluated, such as scanning electron microscopy (SEM) [18,20,21], Fourier-transform infrared spectroscopy (FTIR) [12], thermal analysis as differential scanning calorimetry (DSC) and thermal analysis as thermogravimetry (TGA) [13,20,22]. Aparicio Ardila et al. [21] showed that DSC analyses could detect changes in a geotextile’s molecular structure caused by the material’s decrease in crystallization. Marques et al. [12] verified structural changes in TGA and FTIR analyses of geosynthetics, which could explain alterations in mechanical properties.

Therefore, to contribute to the study of geomat durability, the objective of this paper was to evaluate degradation through thermal and mechanical analysis of geomats exposed to laboratory and field conditions for 74 months. This work is a continuation of the research developed by Vianna et al. [23]. They assessed the performance of 12 bioengineering techniques for controlling erosion in prone slopes of Simplício Hydroelectric Power Plant (HPP) Furnas-Brazil. Thus, in this work, degradation of the permanent section of a three-dimensional polypropylene geomat is evaluated.

## 2. Materials and Methods

### 2.1. Geomat Characteristics

**Figure 1:**
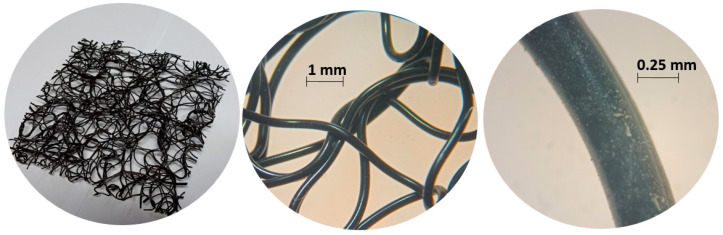
Geomat used in the erosion-control techniques.

The geomat used to control the erosion on the slopes was a three-dimensional geomat manufactured using polypropylene (Figure 1). The physical and mechanical properties of the geomat are presented in Table 1.

### 2.2. Exposure in the Field

**Figure 2:**
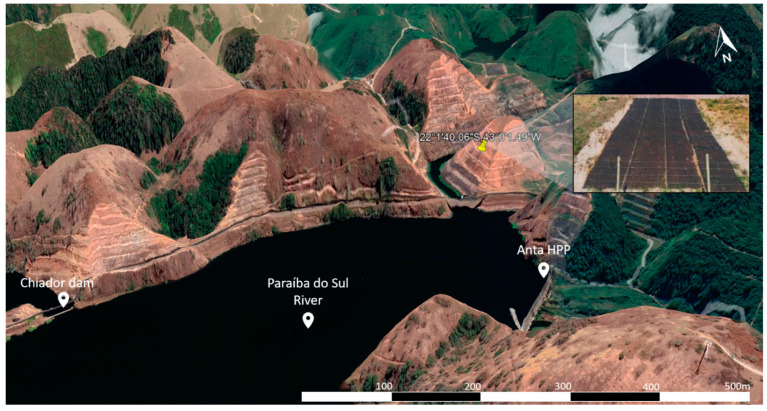
Locations of the experimental units (EUs) in the HPP Simplício (22°1′40.06″ S; 43°0′1.49″ W).

The treatment for erosion control on the slope was installed in an experimental unit located at HPP Simplício. This HPP is located on the border of the Rio de Janeiro and Minas Gerais states in Brazil, specifically in the municipality of Chiador-Minas Gerais, close to the Anta HPP, on the border of the Paraíba do Sul River. The experimental unit is located at coordinates 22°1′40.06″ S; 43°0′1.49″ W (Figure 2). Selecting the place to install the treatment was carried out by analysing satellite images. The treatment was installed in 2016.

**Figure 3:**
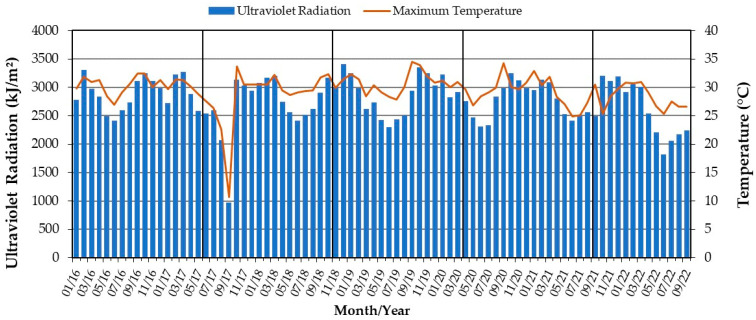
Meteorological information of the site over the study period (January 2016–September 2022).

In this region, the climate has dry winters and hot summers, classified as Cwa according to Koppen climate classification [27]. Figure 3 shows the meteorological conditions of the study area. The geomat exposure period was from July 2016 to September 2022. The National Institute of Meteorology (INMET) provided meteorological data about the exposure period. These data are from the meteorological station located in the municipality of Três Rios in the State of Rio de Janeiro, 22.68 km from the experimental unit. The average annual regional temperature is 29.5 °C, ranging between a minimum of 0 °C in winter and a maximum of 37 °C in summer. The annual precipitation for the study area is around 1512 mm, considering the period of study.

### 2.3. Exposure in the Laboratory

Exposure in the laboratory was carried out using an Equilam UV weathering chamber (model EQUV 003; São Paulo, Brazil) with UVA-351 fluorescent lamps programmed to operate in cycles of 20 h of UV light at 75 °C followed by 4 h of condensation at 60 °C [20]. Two geotextile samples (20 × 30 cm^2^) were exposed for 500 h and 1000 h, respectively. The reason for using these exposure times for UV radiation is that continuous submissions of 500 h and 1000 h can be equivalent to 1 and 2 years of exposure to these materials in an open environment [28], respectively. After exposure, subsamples were taken from each sample to perform mechanical and thermal analysis of the aged material. The effects of ageing were evaluated by comparing virgin (reference) and aged samples.

### 2.4. Mechanical Method

Tensile tests were carried out on the constituent wires of the geomat, different from the traditional (standardised) tensile tests used for uniform geosynthetic materials such as geotextiles or geogrids. Standardised tensile-strength tests do not fully represent the tensile behaviour of a non uniform material, such as a geomat. The tensile tests on the geomat wires were performed with an Emic model DL 3000 universal testing machine coupled with a signal conditioning system and data-acquisition model D4 from Micro-Measurements Vishay Group. A load cell was configured with a maximum capacity of 50 N and a test speed of 20 mm/min [26], in a gripper with 25 mm × 25 mm jaws.

### 2.5. Thermoanalytical Methods

In this study, the thermogravimetry (TG) and differential scanning calorimetry (DSC) methods were used for thermal evaluation of virgin and aged materials.

The TG analysis was performed with Mettler Toledo equipment, model TGA/SDTA 851, with a robotic system to place and remove crucibles. The samples were placed in an aluminium crucible with a mass of around 9 mg, and nitrogen purge gas was used, with a flow rate of 100 mL min^−1^ and a heating rate of 10 °C min^−1^.

DSC analyses were carried out in aluminium crucibles with lids and without holes. The temperature range was the following: (I) cooling from 25 to −70 °C; (II) heating from −70 to 200 °C; (III) cooling from 200 to −70 °C; (IV) heating from −70 to 200 °C and (V) cooling from −200 to 25 °C. These intervals were used to compare samples, verify heating effects and cool all samples. The heating and cooling ratio was 10 °C min^−1^, and the nitrogen purge gas had a flow rate of 50 mL min^−1^. Measurements were obtained utilising Mettler Toledo equipment, model DSC 822, with a robotic system to place/remove crucibles and using samples with masses close to 6.5 mg.

## 3. Results and Discussion

### 3.1. Degradation and Resistance of Polymer Wire

**Figure 4:**
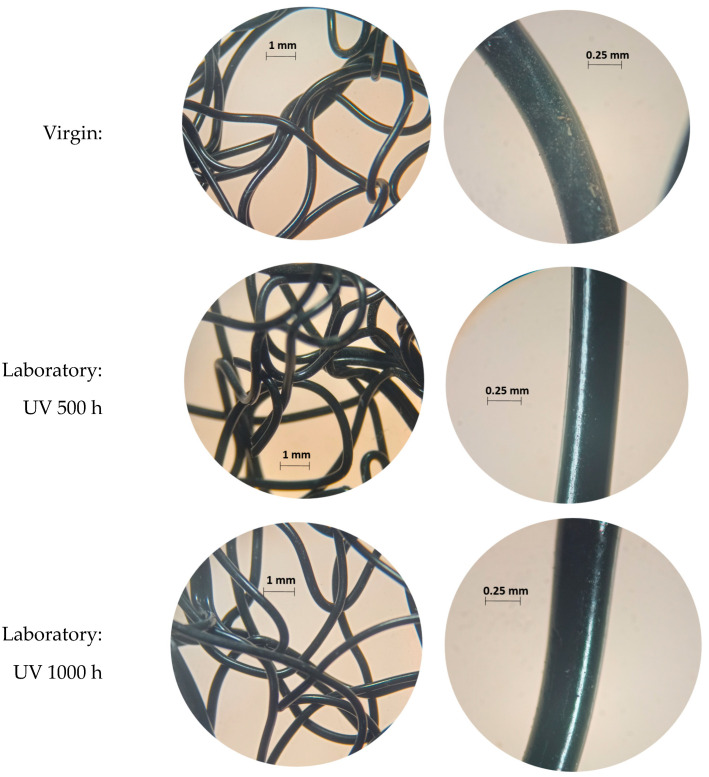

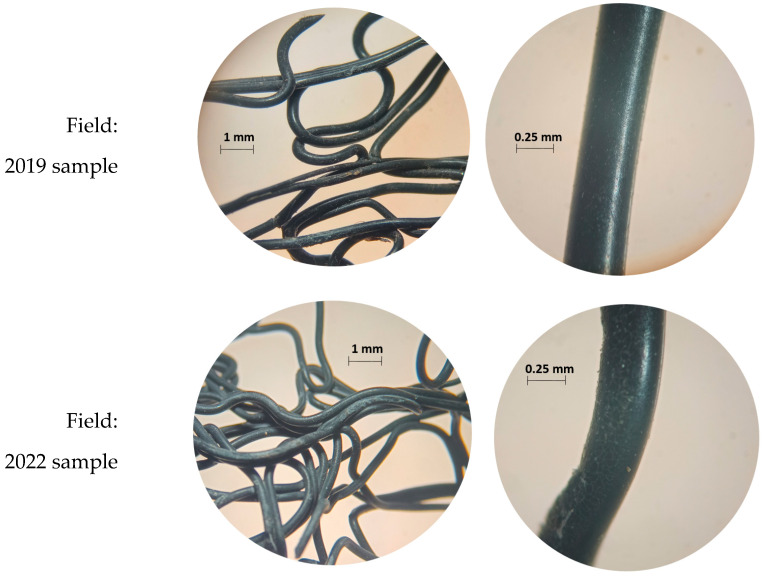
Degraded geomat samples in field and laboratory.

As a physical characterisation, images of the virgin samples were taken, exposed in an ageing chamber and degraded in the field with a Stereomicroscope from Bel Photonics (Piracicaba, Brazil), Model STM Pro, with a 25× magnification lens, as can be seen in Figure 4.

Geomats are three-dimensional and discontinuous materials around the thermal union between the filaments, and it is not possible to carry out evaluations with the wide-width tensile test. Thus, the most viable alternative is the tensile strength of the wires, separately, sampled from the material body.

**Figure 5:**
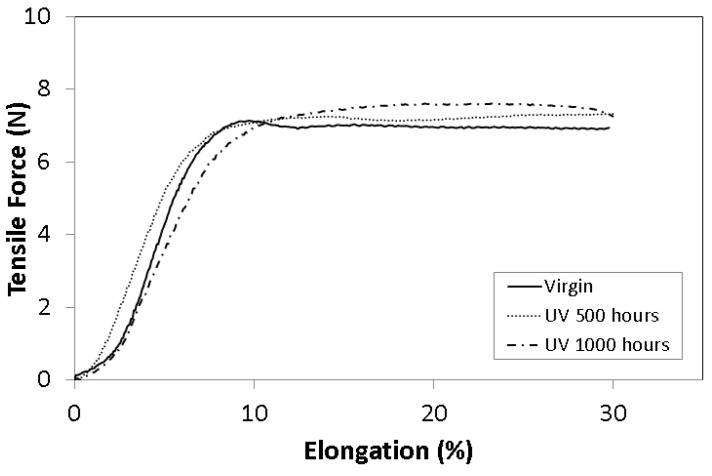

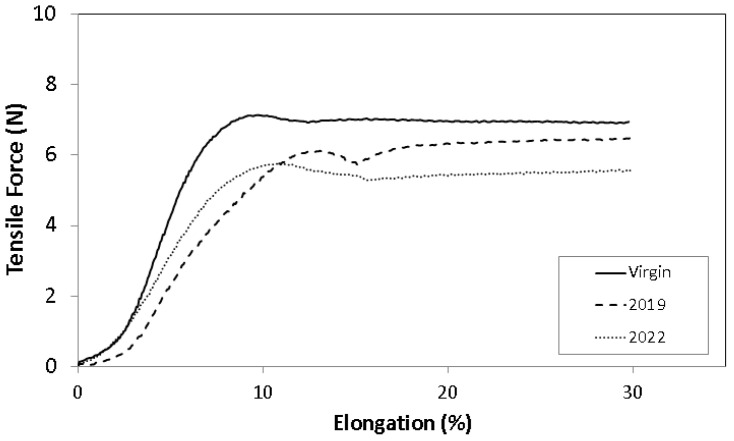
Resistance of geomats degraded in laboratory (UV 500 h and UV 1000 h) and in field (2019 and 2022).

The degradation of the polymer wires was evaluated using a tensile test of the wires, as shown in Figure 5. Tensile tests were carried out on the geomat wires, bearing in mind the mechanical variability, an essential characteristic of this geosynthetic material. The geomat wires exposed to field exposure (in 2019 and 2022) showed less resistance than considered based on ageing in the laboratory (UV), which was to be expected, since the exposure times, to UV radiation, of 500 h and 1000 h are equivalent to after 1 and 2 years of exposure in an open environment, respectively.

### 3.2. Thermal Analyses

**Figure 6:**
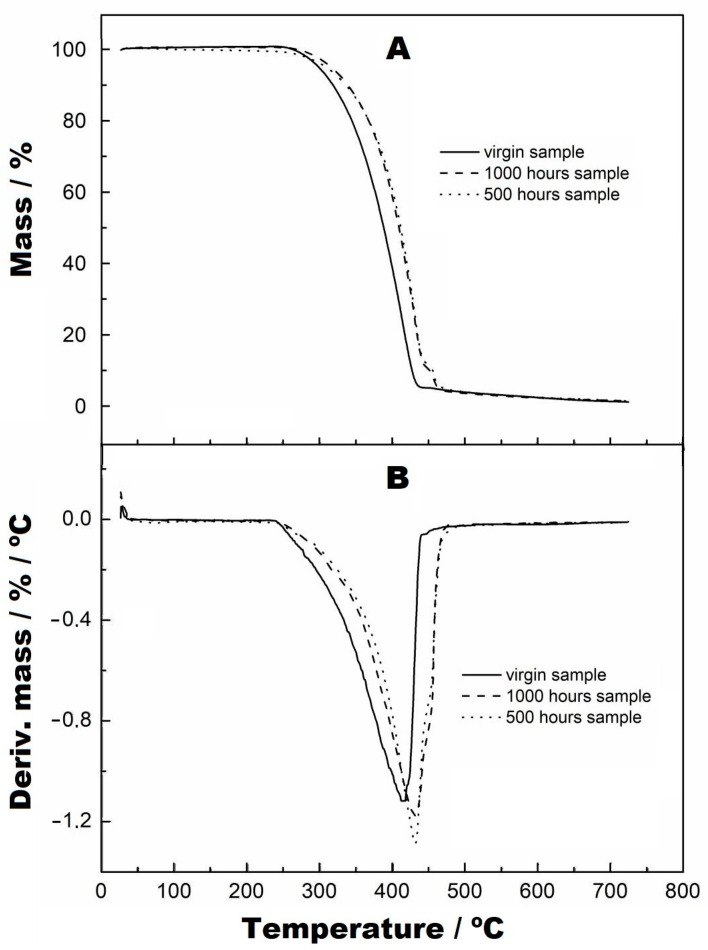
TG curves from virgin, at 500 h and at 1000 h, with a heating rate of 10 °C/min, nitrogen purge gas and aluminium crucibles.

**Figure 7:**
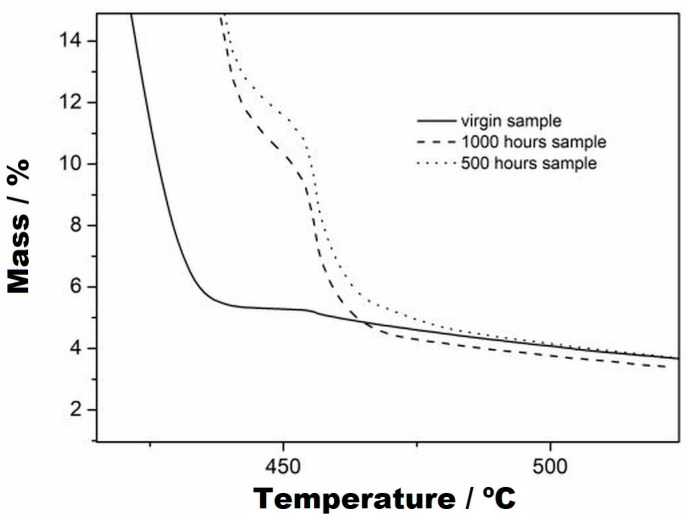
TG curves with zoom applied to the second stage of thermal decomposition, from virgin, at 500 h and at 1000 h, with a heating rate of 10 °C/min, nitrogen purge gas and aluminium crucbles.

**Figure 8:**
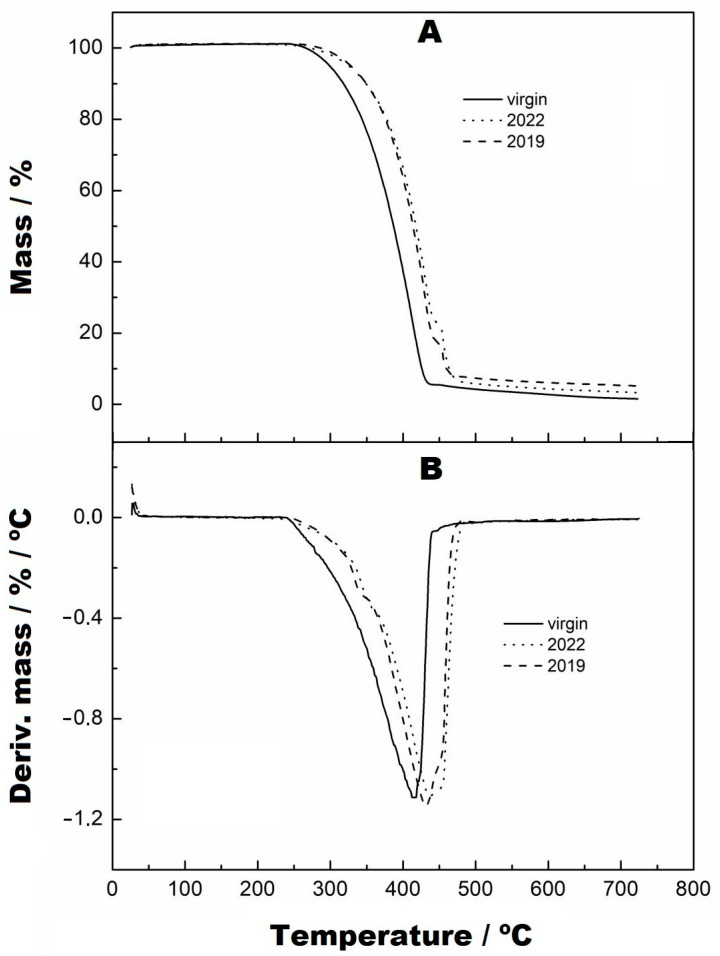
TG (**A**) and DTG (**B**) curves from virgin samples collected from the base at two points on the reservoir margins, with a heating rate of 10 °C/min, nitrogen purge gas and aluminium crucibles.

**Figure 9:**
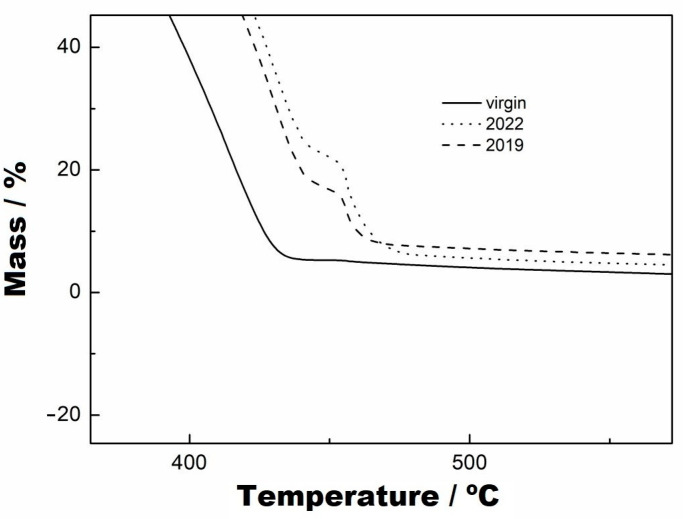
TG curves with zoom applied to the second stage of thermal decomposition, from virgin, 2019 and 2022, with a heating rate of 10 °C/min, nitrogen purge gas and aluminium crucibles.

**Figure 10:**
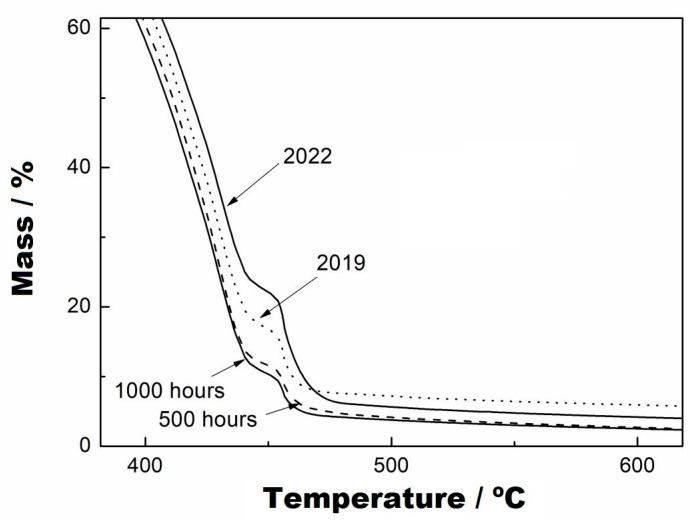
Comparison between TG curves from samples collected and UV radiation conditions.

**Figure 11:**
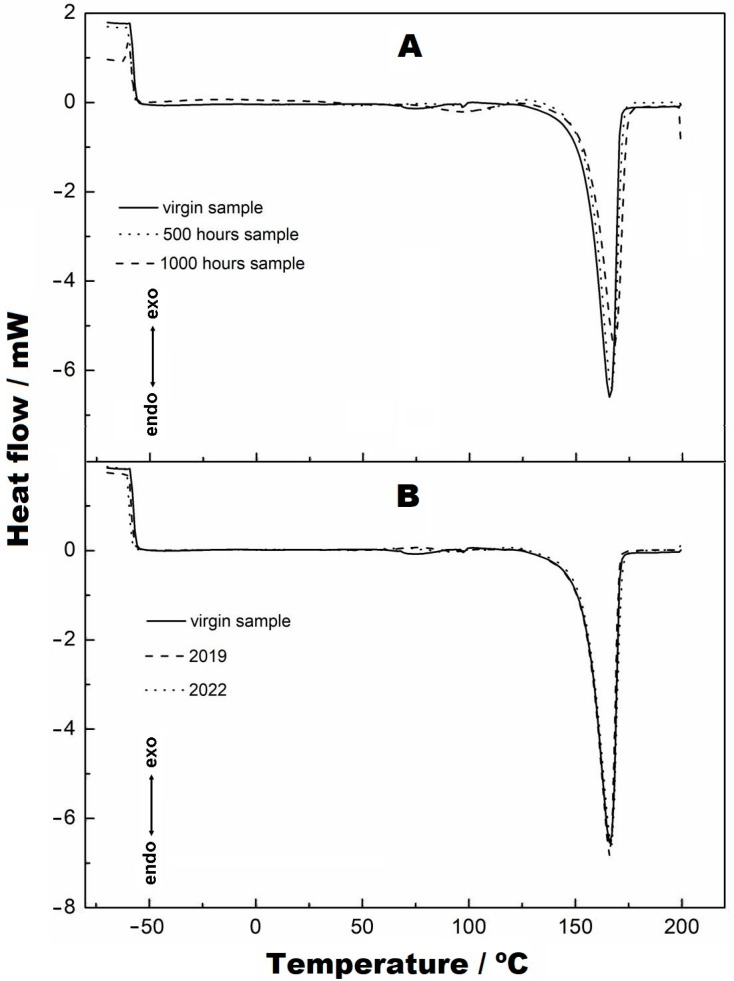
Endothermic curves referring to the melting points: (**A**) virgin, 500 h and 1000 h; (**B**) virgin, 2019 and 2022.

**Figure 12:**
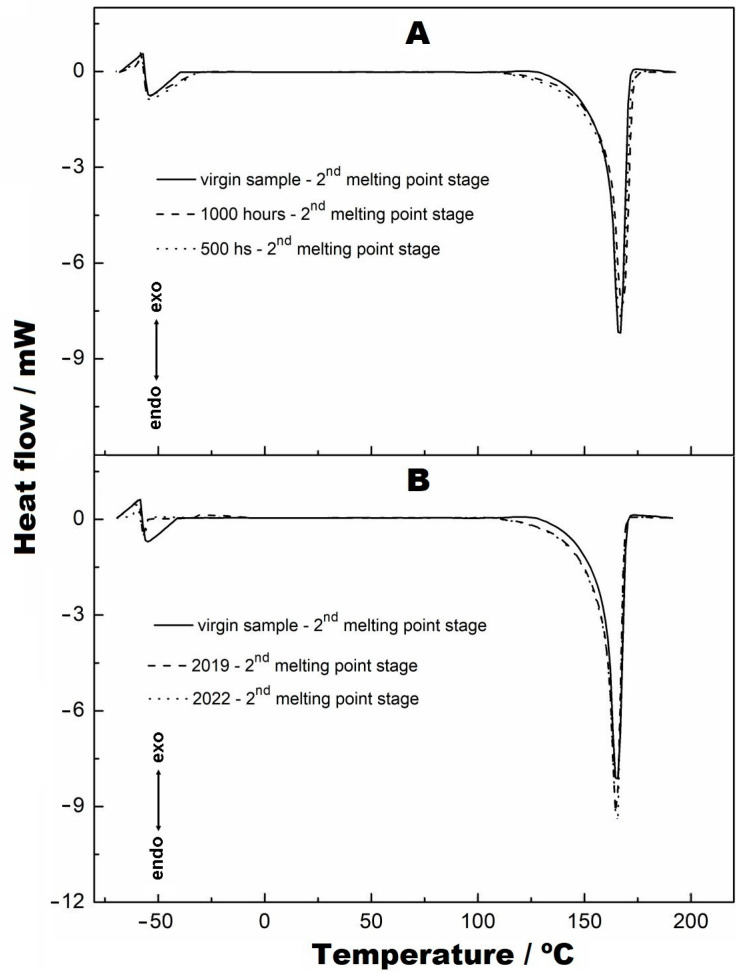
Endothermic curves referring to the second melting points: (**A**) virgin sample, 500 h and 1000 h; (**B**) virgin sample, 2019 and 2022.

**Figure 13:**
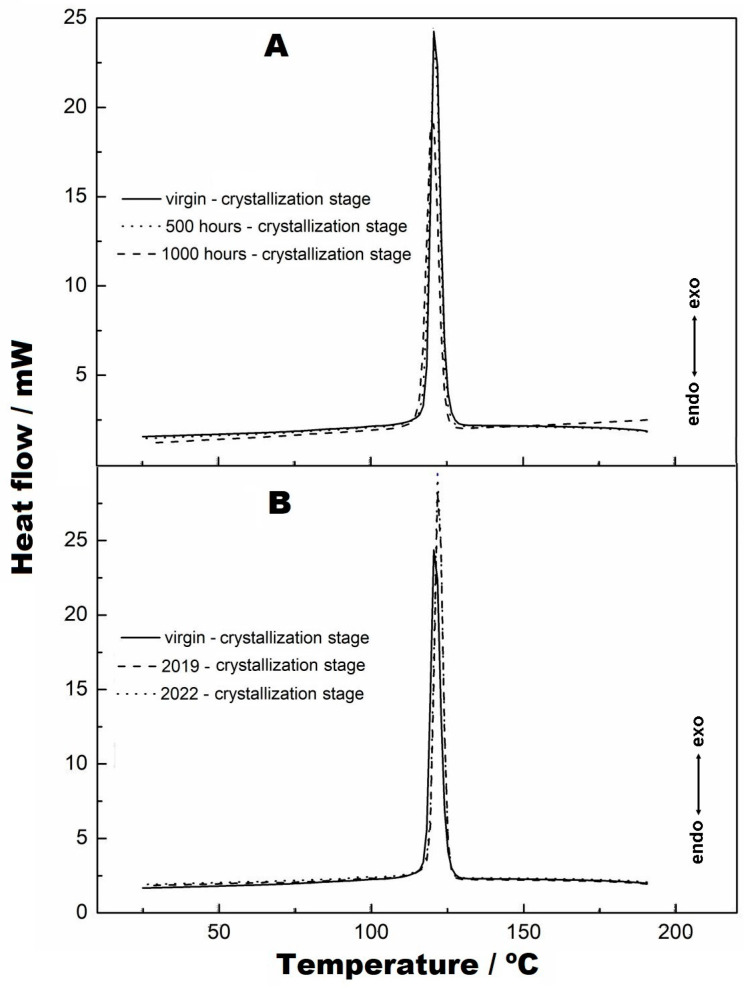
DSC curves of crystallization: (**A**) virgin sample, 500 h and 1000 h; (**B**) virgin sample, 2019 and 2022.

The TG and DSC curves of the virgin sample and the samples degraded in the laboratory (UV radiation) and the field were performed at a heating rate of 10 °C min^−1^. The results obtained with the TG and DSC analyses are shown in Figure 6, Figure 7, Figure 8, Figure 9, Figure 10, Figure 11, Figure 12 and Figure 13. The two samples collected at the base of the HPP Simplício slope were named 2019 (extracted in 2019) and 2022 (extracted in 2022). These samples were compared with the virgin sample and those subjected to UV radiation (in the laboratory).

Figure 6A,B present, respectively, the TG and DTG curves of the virgin sample and of the samples subjected to UV radiation at 500 and 1000 h. In the DTG curves, it was observed that there was no mass variation in these samples up to the temperature of 235 °C. The first mass variation of the virgin sample occurred in a single step up to 445 °C, with a loss of 94.87% of the mass, as seen in the TG and DTG curves (Figure 6A,B). For the samples subjected to UV radiation, it could be seen in the TG and DTG curves that there were two stages of decomposition, where the first occurred at up to 440 °C for both samples, with a mass loss of 86.14%. The second stage of mass variation of these samples can be seen in Figure 7, and the following results were obtained: for 500 h, an interval of 440-481 °C and mass variation of 8.75%, and for 1000 h, an interval of 440–470 °C and mass variation of 8.67%. Furthermore, the two curves were practically coincident in behaviour, which indicates that they had the same decomposition characteristics. However, the virgin sample had a lower decomposition stage than the samples subjected to UV radiation.

The analyses of the samples collected from the two periods are shown in Figure 8 and Figure 9. Figure 8 indicates that the TG curves have two mass variation events, and Figure 9 shows in more detail the second variation stage of the mass, which is similar to that presented by the samples at 500 and 1000 h. The temperature ranges and mass variations, respectively, were: (i) sample collected 1, 235–443 °C/81.67% and 235–443–476 °C/10.15% and (ii) sample collected 2, 235–445 °C/76.90% and 445–482 °C/16.96%. However, in observing the DTG curves, they show that the decomposition occurred from 235 °C and there are two events of mass variation in the collected samples, at the temperature of 334 °C, that are not seen in the TG curves due to their low magnitude because of the use of a percentage scale. Furthermore, the DTG curves show the formation of shoulders just after the maximum point of the second mass variation. It can also be observed that the thermal decomposition of the virgin sample occurred before that of the collected samples, as seen in the TG and DTG curves. At the end of the reaction of these collected samples, it can be seen that both had different formations of residues at the end of the thermal decomposition when compared with the TG curve of the virgin sample.

For comparison purposes between the samples collected and those subjected to UV radiation, Figure 10 shows the overlapping of these curves. As seen in the TG curves, there are events at the mentioned temperatures above that differ from each other by percentage; that is, the samples subjected to UV radiation had the same thermal-decomposition characteristics, while the samples exposed to the medium environment had similar thermal-decomposition-behaviour characteristics but with slightly different percentages. Both show two stages of thermal decomposition, with the ranges of temperature variation and mass loss given above.

Indeed, the time of exposure to UV radiation and also exposure to the environment increased the thermal decomposition temperatures of the samples. This fact does not mean that exposure improves or increases useful life, which is obvious, considering the degradation of the material under the action of UV radiation (either from lamps or from the sun). Degradation caused by UV radiation changes the structure of the molecules, which probably breaks intermolecular or intramolecular bonds. Consequently, there was a change in the thermal decomposition behaviour. Thus, during thermal decomposition, the material formed other stable stages. At the end of the thermal decomposition, the formation of carbonaceous material impregnated in the crucible was observed for all samples.

The DSC analyses are shown in Figure 11, Figure 12 and Figure 13. Figure 11A,B show the endothermic curves referring to the melting points of the virgin sample, at 500 h and at 1000 h and of the samples from collection points 1 (2019) and 2 (2022). The melting points of these samples are coincident, and there is no change in the melting peak of the curves. However, for the samples at 500 and 1000 h (Figure 11A), a transition is seen before the effective melting of the sample occurs, which is attributed to the softening of these materials. This is attributed to small parts of the fibres corrupted by the radiation and fusing earlier than the larger, more structured fibres. Nevertheless, Figure 11B shows the virgin samples, collection 1 (2019) and collection 2 (2022), and it can be observed that the “pre-melt” event is not seen, as observed in the samples at 500 and 1000 h. This fact indicates that there may be two explanations for this occurrence, which must be considered: (i) the samples subjected to radiation were not exposed to the weather, which made them purer than the samples that were exposed, and therefore, the residues of the degradation merged beforehand, and (ii) the fact that the 2019 and 2022 samples were exposed to the external climate, causing the presence of impurities, which were impregnated in the structure, to occur, which did not allow the event (here called the prefusion event) to occur, as the samples exposed to UV radiation did not contain external impurities.

Figure 12A,B show the second melting points of these materials, which were achieved by reheating these materials. As seen in both figures, alterations of the baselines of the DSC curves can be observed before the melting points and at the melting points. This fact has already been verified in the work performed by Valentin et al. [22] and by Lavoie et al. [29]. This is attributed to the effect caused by heating the materials in the first melting process, which “erases” the effects caused by radiation in the materials; i.e., the broken structures were diluted in the melt, given that the melting process caused the transformation of the material to the liquid state. This effect is better understood when the crystallization curves in Figure 13A,B are visualised. It can be observed that the peaks coincide with each other due to the melting process, which transformed the materials from heterogeneous to homogeneous. Crystallization is essential, as it shows that a material remains crystalline and retains its original characteristics. The crystallization curves of the second heating are not shown because they have similarity to those obtained in Figure 13.

## 4. Conclusions

Laboratory simulations are essential for predicting service life and studying the behaviours of various geosynthetic materials. In the present case, this study was carried out to obtain a parameter of the laboratory relationship (exposure in an ageing chamber) versus in the field. However, it was observed that, for periods of exposure of 500 and 1000 h in an ageing chamber and under the mentioned study conditions, the same degradation behaviour was not obtained when compared with the materials exposed in the field. This fact could be attributed to field-climate variations during the study period, which were different from what was used in the ageing chamber, or even the fact that the exposure periods of 500 h and 1000 h were incompatible with the field samples’ collection period. More extended periods are needed to reach similar levels of behaviour.

Based on the mechanical and thermoanalytical behaviours of the polypropylene geomats exposed in the field and laboratory-tested in this study, the following conclusions are highlighted:

- The geomat wires exposed in the field showed a more significant decrease in resistance when compared to the results of the samples exposed in the laboratory. This indicates that exposure in the field is more aggressive than exposure to UV radiation in a laboratory.
- In the samples collected in the field, it was observed that the TG curves showed thermal decomposition of the virgin sample before the collected samples, as seen in the TG and DTG curves. Contrary to what was observed in the samples exposed in the laboratory, where the virgin sample showed a lower thermal decomposition stage (as seen in the TG curves) than the samples submitted to UV radiation, the samples exposed to UV radiation (500 h and 1000 h) presented the same decomposition characteristics.
- The exposure times in the laboratory and the field increased the thermal decomposition temperatures of the samples. This indicates that degradation caused by UV radiation (in the field and the laboratory) alters the structure of molecules, generating disruption of intermolecular or intramolecular bonds and consequently changing decomposition behaviour. Thus, during thermal decomposition, a material goes through other, more stable stages (which undergo other stages of decomposition). This was verified with the formation of carbonaceous material impregnated in the crucibles during the TG tests.
- From the DSC analyses, the samples showed similar behaviours for the melting peaks.
- Evaluation of the wires of the geomats was presented as an alternative to analysing the tensile strengths of discontinuous geosynthetic materials such as the geomats.

## Figures and Tables

**Table 1:** Geomat properties.

Property	Test Method	Average Value
Mass per Unit Area (g/m^2^)	ABNT NBR ISO 9864 [24]	505
Thickness (mm)	ABNT NBR ISO 9863-1 [25]	9.1
Tensile Strength per Unit (kN/m)	ABNT NBR ISO 10319 [26]	0.92
Elongation at Break (%)		125.7

## Data Availability

Data sharing is not applicable to this article.
